# Commercialization Report: DoseOptics LLC

**DOI:** 10.1117/1.BIOS.2.4.040502

**Published:** 2025-11-24

**Authors:** Brian W. Pogue

**Affiliations:** Thayer School of Engineering at Dartmouth, Hanover, New Hampshire, United States

## Abstract

This community report discusses the experience of navigating the complex path of commercializing biomedical technologies, offering lessons for the scientific community and industry stakeholders alike.

## Company Overview and Founding Team

DoseOptics LLC was founded ten years ago to bring real-time visualization to radiation therapy. The company was co-founded by William Ware, now CEO, and Brian W. Pogue, who serves as president. The founding team quickly expanded to include Venkat Krishnaswamy (CTO), Petr Bruza (head of hardware), and Michael Jermyn (head of software), forming a multidisciplinary leadership group with expertise in business, imaging, software, optics and clinical technology.

## Technology Description

**Fig. 1:**
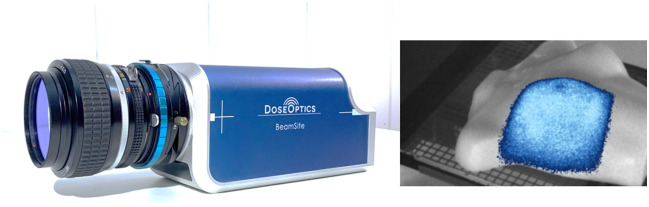
DoseOptics BeamSite camera technology (left) leverages detection of Cherenkov light (right)—emitted when high-energy x-ray beams interact with tissue—to visualize treatment dynamics in real time. Image courtesy of DoseOptics.

DoseOptics manufactures specialized cameras and software systems that detect and display radiation therapy as it is delivered to patients (the DoseOptics BeamSite camera is depicted in Fig. 1). The technology leverages detection of Cherenkov light—emitted when high-energy x-ray beams interact with tissue—to visualize treatment dynamics in real time. Sensitive cameras, synchronized with the x-ray pulses of clinical linear accelerators, capture this light coming from the patient’s skin, allowing therapists to view the beam shape directly at the console during treatment.

## Clinical Need and Market

Radiation therapy is a complex procedure requiring millimeter-level precision across daily treatments that can span several weeks. During beam delivery, therapists must leave the room, making direct visual observation impossible. DoseOptics addresses this challenge by enabling visual confirmation of treatment delivery, enhancing safety and allowing for intuitive verification. Since patients often return 30-40 days in a row, if errors are detected, corrections can be made in subsequent sessions to ensure accurate targeting of radiation.

## Commercialization Stage and Sales

DoseOptics reached the revenue positive generation stage this year, having been entirely funded prior to this through the Small Business Innovation Research (SBIR) program. The company has partnered with a strategic commercial entity that leads sales and marketing efforts, with the product labeled as DoseRT™ or BeamSite™ systems. The product is installed in approximately twenty cancer treatment centers, with the first initial sales prior to this year in clinical research. Expansion into clinical service markets is underway, targeting early technology adopters in academic and advanced radiotherapy clinics. Sales cycles in this high-tech field typically span up to a year, with predictions for sales next year double this current year.

## Competitive Landscape

The technology is unique, with few direct competitors. While some companies offer surface guidance systems for 3D patient mapping, they do not provide imaging of the radiation beam itself. DoseOptics’ ability to visualize beam delivery in real time offers a distinct advantage in treatment verification.

## University Support and IP Licensing

The initial technology was invented and patented at Dartmouth and was exclusively licensed to DoseOptics in exchange for a small equity stake, and the Dartmouth Cancer Center and Department of Radiation Oncology at Dartmouth Health were major partners in testing and development of the technology. This partnership was essential in the company’s early years, enabling access to intellectual property with minimal upfront costs. The university remains involved in additive patents and was a key partner in company formation.

## Funding History

Initial funding for DoseOptics had come exclusively from SBIR grants. The company did not pursue venture capital due to insufficient SBIR support over the 8–10-year development timeline. In 2023 a strategic commercial partner company was found to invest and develop the product and become the lead on all clinical sales and marketing.

## Regulatory Pathway

DoseOptics pursued FDA clearance through the 510(k) pathway, establishing substantial equivalence to existing technologies. Subsequent versions of the system have also received 510(k) clearance, building on the initial approval.

## Lessons Learned

The team found that obtaining 510(k) clearance was manageable, especially with the help of experienced consultants and strategic hires. While consultants can be costly, their involvement was short-term and highly effective. Developing a robust quality management system, though tedious, was essential for FDA approval, and having a dedicated experienced expert in this area is critical. Since initial approval, multiple subsequent 510(k) clearances have been obtained, making the produce more commercializeable with stronger indications for use in the clinic.

## Supplemental Information

The full interview with Brian Pogue is available in podcast form at *Biophotonics Discovery: The Podcast*, available wherever you get your podcasts—YouTube, Spotify, etc.

